# Infusion pumps and red blood cell damage in transfusion therapy: an integrative revision of the academic literature**^1^**

**DOI:** 10.1590/1518-8345.1155.2763

**Published:** 2016-08-15

**Authors:** Ana Maria Miranda Martins Wilson, Maria Angélica Sorgini Peterlini, Mavilde da Luz Gonçalves Pedreira

**Affiliations:** 2 MSc, RN, Escola de Enfermagem, Universidade de São Paulo, São Paulo, SP, Brazil.; 3 PhD, Associate Professor, Escola Paulista de Enfermagem, Universidade Federal de São Paulo, São Paulo, SP, Brazil.

**Keywords:** Nursing, Infusion Pumps, Erythrocytes, Hemolysis

## Abstract

**Objectives:**

to obtain information from scientific literature concerning infusion pumps used in administering erythrocyte (red blood cells) and to evaluate the implications in the practical use of this equipment by nurses when conducting transfusions.

**Method:**

an integrative revision of the following scientific databases: Pubmed/Medline, Scopus, the Virtual Library for Health, SciELO, Web of Science and Cochrane. The following descriptors were used: "infusion pumps", "blood transfusion", "transfused erythrocyte" and "hemolyis". There were no restrictions on the scope of the initial data and it was finalized in December 2014. 17 articles were identified in accordance with the inclusion and exclusion criteria.

**Results:**

all of the publications included in the studies were experimental in vitro and covered the use of infusion pumps in transfusion therapy. A summary of the data was presented in a synoptic chart and an analysis of it generated the following categories: cellular damage and the infusion mechanism.

**Conclusion:**

infusion pumps can be harmful to erythrocytes based on the infusion mechanism that is used, as the linear peristaltic pump is more likely to cause hemolysis. Cellular damage is related to the plasmatic liberation of markers that largely dominate free hemoglobin and potassium. We reiterate the need for further research and technological investments to guide the development of protocols that promote safe practices and that can contribute to future clinical studies.

## Introduction

Blood transfusion is a therapeutic technology that is commonly used in clinical practices in many different health establishments. Approximately 85 million blood transfusions are done annually in the world, with 15 million of them being carried just in the United States^1^. In Brazil in 2014 3,127,957 transfusions were carried out, in which concentrated erythrocytes (CH) were the blood components most used, covering 57.98% of all transfusions conducted in outpatient units and hospitals in the country^2^. 

An indication for a CH transfusion is both clinical and laboratorial which is based on hemoglobin and hematocrit levels as well as any signs and symptoms presented by the patient^1^ ^,^ ^3^.

The implementation of transfusion therapy requires the use of an integrated multidisciplinary team where the following occurs: obtaining blood donors, collection, processing, quality control, distribution, the therapy is prescribed for someone, the transfusion takes place and monitoring the clinical responses^3^ ^-^ ^4^. The role of nurses is fundamental in this process according to the Nursing Federal Council Resolution (COFEN) nº 306/2006 which provides directives on the procedures to be used by nurses in hemotherapy. Nurses are professionally qualified to plan, execute, coordinate, supervise and watch patients during their transfusion therapy^5^.

In relation to the administration of blood components, nurses are required to use infusion devices that are on the market that present differences concerning: control methods for quality, price, presentation and recommendation of use. The national practice for transfusions involves the use of blood transfusion products that work through the use of gravity with a manual flux control system^4^ ^,^ ^6^. 

The infusion pumps (BI) are devices that regulate the flux of liquid administered under positive pressure to the patient. They are used in intravenous therapy in different areas of health care^7^ ^-^ ^8^. Currently BIs are gradually being introduced into the market to be used in blood transfusions. 

With reference to flux control, the BIs can be classified as: volumetric infusion pumps, Flow meters and syringe pumps. Volumetric equipment is a type of device that controls the liquid to be infused in volume by unit of time through programming the flow done by the operator. This then controls the syringe pumps. Flow meters or drips work through permitting flows and are also controlled by an operator. However the control of the infused volume is carried out through the counting of drips per unit of time using an electronic sensor. Syringes pumps are instruments in which the volume administered to the patient is stored through the use of one or more syringes which is pushed by a movable piston controlled by equipment. The operator can select the flow rate and can indicate volumes through units of time on the equipment. Barring the BIs syringe pumps, the other devices essentially work through the use of peristaltic mechanisms and cassettes^7^ ^-^ ^8^.

The peristaltic mechanism permits the infusion of liquid through forcefully pushing a part of it from the equipment through which the liquid passes. This can be done through two methods: the peristaltic linear method or the rotatory method. The two methods are different in that one works through the use of wave movement and the other works based on compression on the linear plaques or gyrating rollers respectively. This results in pushing the liquid from the bottle that has the solution to be infused in the circulatory vein network in the patient^7^ ^-^ ^8^.

The cassette mechanism involves infusion through the use of pistons. Such devices have cassettes in side of them that are generally inserted into the center of the equipment. The pistons, upon being actioned, move in and out of the cylinders that are contained in the cassettes. The internal movement pushes the liquid in the direction of the patient, whilst the external movement drains the liquid from the bottle in order to refill the cassette. Additionally there can be diaphragm mounted on the movable pistons inserted in the cassettes. The engine transmits a movement to the pistons that move in and out of the bottles, compressing the siliconized, diaphragmatic membrane permitting it to be filled or to release the liquid. When the pistons go into the cylinders, the liquid is pushed in the direction of the patient and when the pistons move out, the liquid is sucked up from the container that has it, allowing the cassettes bottles to be refilled^7^ ^-^ ^8^. 

Although there are innumerable advantages in relation to the safety of the patient through the use of BI in intravenous therapy such as the alarms that it has, the control of the infused volume and providing the adequate time for the liquid to be administered, there is still some uncertainty on their use in transfusion therapy due to the effects of the infusion mechanism on the erythrocytes which may result in hemolysis^9^ ^-^ ^10^.

Patients that are transfused with erythrocyte hemolysate, aside from receiving low levels of functional hemoglobin, can be subject to deleterious effects to the organisms through the presence of freed biomarkers when hemolysis occurs^9^ ^-^ ^10^. With consideration for the above, the guiding question for this study was: If one transfuses erythrocytes using BI, will this result in cellular damage and hemolysis?

## Objectives

To obtain information from scientific literature concerning the effects of infusion pumps used in administering erythrocyte (red blood cells) and to evaluate the implications in the practical use of this equipment by nurses when conducting transfusions. 

### Material and method

We undertook an integrative revision of the relevant literature. We collected data from secondary sources based on a list that we had sourced out. We then analyzed the data obtained in a systemized manner.

The study had six steps: 1) An identification of the theme and a selection of the theory or questions for the research, 2) establishing inclusion and exclusion criteria for the study, as well as conducting searches in the literature, 3) defining the information to be extracted from the research that was selected, 4) categorization and evaluation of the included studies, 5) interpreting the results and 6) summarizing the knowledge obtained ^11^ ^-^ ^13^.

This research method brings scientific rigor to clinical practices. This permits the inclusion of different types of studies which allows for the following: the maximization of research, critical evaluation and the summarization of evidence that is obtained based on the theme ^11^ ^-^ ^13^. 

In order to select the articles, we used the following portals and databases: *U.S. National Library of Medicine* (PUBMED), Virtual Library for Health (BVS), SciELO, *The Cochrane Library* (Cochrane), *SCOPUS and ISI Web of Science.* When using the SciELO database, the descriptors were researched using the basic (not advanced) research field.

In the Science of Health (DECS) and *Medical Subject Headings Section* (MESH) we used the following descriptors in response to the research question: "*infusion pumps*", "*blood transfusion*", "*erythrocyte transfusion*" and "*hemolysis"* . In our searches we used the words AND and OR. 

Our research covered all of the articles published up until the 31 December 2014. The inclusion criteria for the publications were the following: 1) articles published in Portuguese, English and Spanish; 2) complete articles that covered the use of BIs in transfusion therapy; 3) any of relevant study. The exclusion criteria was: 1) Opinions from specialists, chapters from books, summaries of journals, patents and editorials, 2) articles that described cellular damage that occurs when using extracorporeal circulation devices and those which provide circulatory assistance with oxygen for extracorporeal membranes (ECMO).

**Figure 1:**
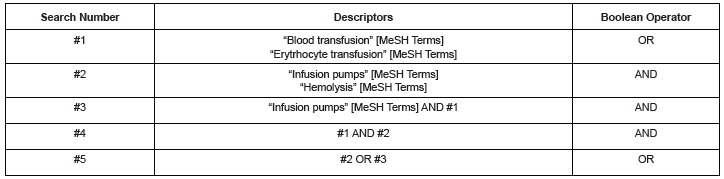
Search Strategy in the Pubmed database- Sao Paulo, 2014

The strategy used to find the articles was modified based on each the database due to the different ways to access each of them. As a guide, we used the research question and the inclusion criteria that had been previously defined. Figure 1 shows the strategy used for the searches done in the Pubmed database. This strategy was used in analyzing the other databases.

### The Procedures for collecting the data

Initially we looked at the title and the summary of the studies to check that they met the inclusion criteria. The next step was to analyze the complete article that was selected. We needed to see whether the study was pertinent to our study. The publications that did not have the complete text were solicited through the *comut*/SCAD. Data collection forms were designed by the researchers and were adapted to the objectives of the research, having the following items: identification of original article, methodological characteristics, an evaluation of the methodological rigor used, interventions and the main results found.

The results were presented in a descriptive way permitting anyone to evaluate the applicability of our integrative revision. It would also help them in making decisions in clinical practices related to transfusions and it would show any knowledge gaps. This could help in developing and enhancing future research. 

### Procedures for analyzing the data

**Figure 2:**
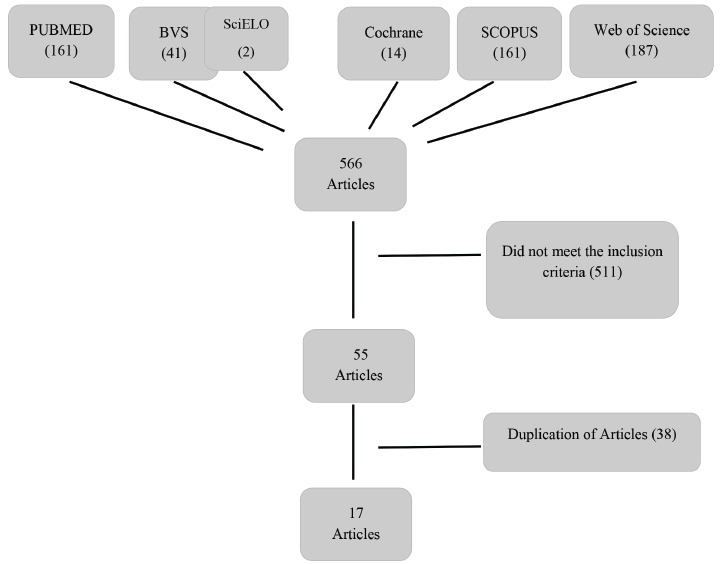
Flow chart of selection of the articles for the database

From the six databases included in the study, we managed to obtain a total of 566 articles. Of these, 511 fell into our inclusion criteria and our proposed objectives, ending in 55 articles. From this total, 38 were taken out as they were repeats from the databases, which left 17 articles (figure 2). 

All of the studies that were included were experimental designs in vitro. No classification of the levels of the evidence was carried out as they were considered to be pre-clinical studies.

Further on in the research, the studies were analyzed and grouped based on their similar content with two categories to be analyzed: the mechanism of the BI and the cellular damage. 

The presentation of the results from the data that was obtained, was presented in a summary chart which had the following information: information on the authors, the objective of the study, type of study, results, conclusion and the implications in the practices of transfusions done by nurses. This last element had relevant points that were summarized from each article that we analyzed that covered the use of infusion pumps in transfusions done by nurses. It was noted that in some places the aforementioned devices were used in blood transfusions.

## Results

Out of the 17 articles that we found, the majority were in English being: 15 (82%), 5.9% were in Spanish and 5.9% were in Portuguese. With reference to the where the studies had been conducted, 10 (58.9%) of them had been produced in the United States, one (5.9%) in England and the remaining six (35.2%) were produced in the following countries: Brazil, Spain, Australia, Denmark, Holland and Switzerland. Amongst the selected studies, the oldest was produced in 1981 and the most recent in 2011.

In relation to the design of the 17 studies selected, all of them were experimental having simulations of the procedures in laboratories. With reference to the listed categories corresponding to the infusion mechanism, we analyzed the peculiarities in relation to blood transfusions and the chances of cellular damage due to the mechanical force of the equipment. In the cellular damage category, we grouped together related variables to the blood components and cellular damaged markers.

**Figure 3:**
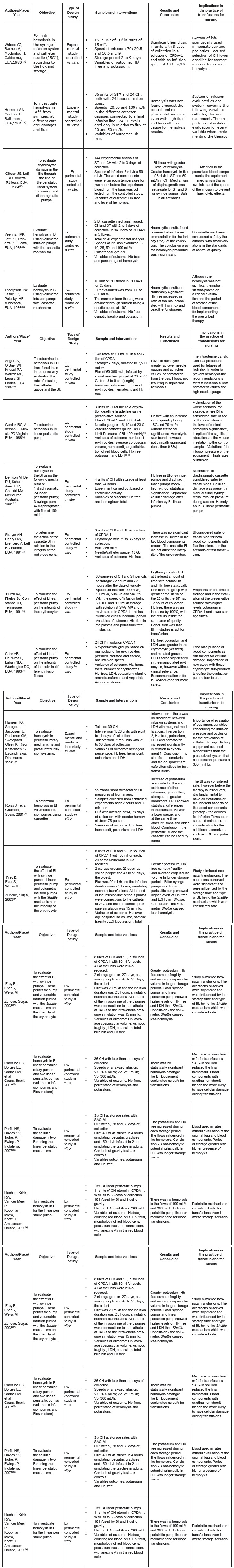
Summary of the studies that were analyzed with reference to the authors, the objective of the study, the results, type of study, results and conclusion and implications for practices in nursing.

Figure 3 is a presentation of a summary of the studies identified and included in the present integrative revision and our principal results. 

* Gauge; † Concentrated red blood cells; ± Milliliters; § Milliliters per hour; | | Hemoglobin; ¶ Citrate phosphate dextrose adenine; ** infusion pumps; †† Blood total; ±± Dose of absorbed radiation§§ Millimeters of mercury; | || | Lactate Dehydrogenase; ¶¶ Saline-adenine-glucose-mannitol.

### Infusion pump mechanisms

Amongst the 17 studies that were selected, we identified 40 types of infusion systems used in transfusion therapy with 39 (97.5%) being BI and 01 (2.5%) being a pressure system. Some work opted for analyzing just the infusion mechanism in isolation or the different manufacturers while others evaluated the differences between the mechanisms. 

The studies covered volumetric infusion pumps, Flow meters and syringe pumps that, in one study, the type of devices were not described. Of the 39 that were described, volumetric infusion pumps were described the most (32 or 82.0%). The next were 5 syringe pumps (12.8%) and lastly 2 flow meters (5.2%).

Amongst the total number of pumps described, the following infusion mechanism were found: five (12.8%) syringe pumps, nine (23.1%) cassette types, 22 (56.4%) linear peristaltic pumps, two (5.1%) rotary pumps and one (2.6%) using the shuttle mechanism.

With reference to the infusion mechanism, the linear peristaltic pump was the type that provoked the most amount of hemolysis. It was stated that hemolytic events could be predicted according to 10 (76.9%) publications of the 13 that were studied covering linear peristaltic pumps. Others studies state that the BI that is considered the safest for therapeutic transfusions is the volumetric infusion pumps using the cassette mechanism. This was mentioned in four studies (or 66.7% out of six) with results that showed how safe it was.

Simulation studies were done with reference to infusion flux based on clinical practices for transfusions done in pediatrics/neonatal care and for adults. The speeds of infusion varied from five mil liters per hour (mL/h) to 999mL/h.

In relation to infusion pressure, two of the studies (11.8%) covered an inflatable pressurized device with a pressure gauge. The other was able to obtain the maximum pressure for linear peristaltic pumps^20^ ^,^ ^25^. 

### Cellular damage 

Out of the 17 studies, the blood components which were prevalent were the CH in 10 of them (58.8%). One (5.9%) only used the ST and six (35.3%) evaluated both blood products in the experiments.

In relation to the preservative solution, eight (47.1%) used the citrate phosphate dextros and adenine solutions (CPDA-1). Four (23.5%) used additives based on mannitol. Four (23.5%) did not specify the preservative solution used and one (5.9%) analyzed samples of both solutions. None of the publications focused on the propensity for hemolytic effects to occur based on the preservative solution. Only two studies suggested (11.8%) that solutions with additives reduced the final hematocrit in the bag. This in turn reduces the viscosity of the blood components.

In relation to storage time, the time period for storing hemolysis varied from 24 to 44 days. In eight studies (47.1%) there was a description of a lot of hemolysis when the erythrocytes were near to expiration^16^ ^,^ ^18^ ^,^ ^26^ ^-^ ^27^ ^,^ ^29^.

In the life span of the hemolysis there is a liberation of hemoglobin in the plasma. There is also an increase in potassium and lactic dehydrogenase (LDH) amongst other biomarkers. In the selected article, the integrity of the cells were analyzed through variables of outcome such as free hemoglobin, hematocrit, potassium, LDH, percentage of hemolysis, a wide distribution of red blood cells, average corpuscular volume, the number of erythrocytes, alanine aminotransferase and aspartate aminotransferase. Free hemoglobin was the biomarker most present in all of the publications. Potassium was the second hemolysis marker that was most prevalent being described in 11 (64.7%) of the studies included. Of these, nine (53.0%) related to the increase in potassium at the longest storage period for the hemotherapeutic product.

With reference to establishing hemolysis as the results of the action of the infusion system in the cells, 10 (58,9%) articles covered this area. Four publications (23.5%) noted significant statistical alterations in the outcome variables. However they opted on determining the presence of hemolysis being clinically significant for values where the level of hemolysis was above 0.8%^18^ ^,^ ^20^ ^,^ ^25^ ^,^ ^28^.

None of the publications mentioned the clinical consequences of cellular damage and the liberation of biomarkers to patients.

## Discussion

The studies selected in the literature show that alterations in the integrity of the erythrocytes can occur when CH and ST are transfused by BI. This is that case for the action with the infusion mechanism and the variables related to the equipment such as flux. 

All of the evidence found in the study came from simulations done in laboratories. Humans were not used in the simulations. These studies were called pre-clinical studies. Publications that rate the level of scientific research on a scale of I to VIII with level I covering systematic meta-analysis revisions which are considered the best evidence, refers to pre-clinical studies with animals. In vitro studies are considered to be VIII on the scale^31^. However they are considered fundamental evidence for investigating theories which can be subsequently evaluated and implemented in future clinical studies. 

Even with a low level of evidence in the in vitro studies, it is still possible to evaluate the methodological rigor of the publications from the way how the studies have been designed, with 16 (94.1%) covering controlled evaluations and 01 (5.9%) covering controlled randomized studies. 

Cellular damage of the red blood cells during the period of the extravascular hemolysis can have damaging clinical consequences for the patient. This is because low levels of functioning hemoglobin are produced and renal problems can occur (such as hemoglobinemia, hemoglobinuria and acute renal problems). There can also be alterations in substances that point to hemolysis such as LDH, haptoglobin and potassium^9^ ^-^ ^10^ ^,^ ^32^. 

Free potassium in the plasma can bring about adverse events for the patient such as arrhythmia and even sudden death. Other correlated studies increased the level of potassium with an increase in storage time and the preservative solution in the collection bag^33-35)^. There is evidence of the occurrence of hyperkalemia and even heart attacks when there are transfusions with CH after long storage periods^33^ ^-^ ^35^. The concentrations of potassium in the stored blood increase about 1 milliequivalent (mEq) per day^9^. However none of the publications that were selected touched on the clinical consequences of cellular damage to patients because they mainly dealt with in vitro studies. In spite of this, they placed a lot of emphasis on the biomarkers as a consequence of cellular damage and hemolysis. 

Nowadays, national agencies, Europeans and North Americans establish a maximum level of hemolysis at 0.8% until the last storage day. This is obligatory in the control of the quality of blood banks^36^. In some included studies the authors designated hemolysis through alterations in the markers after the experiments. Others adopt a reference value for the level of hemolysis at 0.8% for clinical relevant hemolysis. The value designated to control the quality of the CH in blood banks in Brazil is defined by the Resolution from the Governing Collegiate (RDC) Number 34, 11 June 2014. It stated that the acceptable hemolysis is a maximum of 0.8% at the last time period for storage which is about the 35th day in conservative solutions that have CPDA-1^37^. 

Another point that was explored as a factor in damaging red blood cells, was the storage time for ST and CH. Some research noted that red blood cells at the last time period for expiration are very fragile and are susceptible to hemolytic effects. The solutions commonly used CPDA-1 that conserves the CH for 35 days and the additives solutions that preserve erythrocytes for 42 days. Publications suggest that the preservative solutions saline-adenine-glucosemannitol (SAG-M) reduces the final hematocrit for blood components, however further research should focus on the differences between preservative solutions, hematocrit and blood components such as red blood cells leukoreduced, washed and radiated^19^ ^,^ ^28^.

The linear peristaltic mechanisms were the most susceptible at producing hemolysis, according to the articles. 13 studies were evaluated in this revision. 3 (23.1%) of them considered it to be a safe mechanism for transfusions. The flow meters BIs mentioned in two publications are not currently recommended for intravenous therapy because they require electronic sensors that count the drips to measure and administer the volume of liquid. However it does not consider the viscosity, density, superficial tension and solution temperature which are important determinants for measuring the drips to be administered^6^..

The volumetric mechanism with the cassette is efficient and excellent for intravenous therapy. This is because it has little interference with the mechanical force of the BI on the fluid to be administered. In the articles included in the present revision, it was identified as a safe mechanism for blood transfusions^(6,17,21-22.26)^.

An English studied noted that hemolysis is caused by multi factors associated with the increase in hematocrit, storage time for the blood components and the pressure placed on the red blood cells^9^. The manufacturers ought to pay attention to the international standard in the *International Organization for Standardization* (ISO) 1135-4^38^, that determines the maximum pressure level for infusion at 40 kilopascal (kPa)^9^ ^,^ ^25^ ^,^ ^38^. No publication stated the value of the infusion pressure as a possible factor for cellular damage. They only stated the pressor variation in the equipment in high infusion fluxes^20^. Aspects related to accessories to infusion pumps such as catheters, were described and evaluated only in conjunction with the infusion system. This gave us inconclusive and conflicting results. 

The linear peristaltic pumps are the most common pumps in the health care system in the country. They have advantages in comparison to the cassette system, such as similar alarms and their accessories are less onerous^6^. More studies in the linear peristaltic pumps should be done to established standards for its use. The studies should also cover: infusion pressure and occlusion, defining the worst case scenario, fluxes that mimic the practice of transfusions, temperature control during the procedure and a wide analysis of biomarkers for evaluation of hemolysis which has consensus in the literature.

We reiterate the importance of institutional protocols for blood banks and assistant units that ensure safety in the transfusion process to prevent untoward events. For example there can be double checks of the blood component data and the individual who will receive the blood. Health and safety analysis of the equipment can be conducted for transfusions in institutions. There can also be: more improvements made in the technology used and more visual inspections of the collection bags because this visual check may be useful in detecting hemolysis (note this has not be proven)^39^.

Multi professional teams need to do the following to implement the use of this transfusion therapy: prescribe the blood product, plan the installation of the transfusion therapy, choose the catheter and the accessories for adequate infusion, obtain an access route, technical installation, monitoring patient responses, infusion control (time, volume, adverse reactions), prevention of complications and constantly monitoring the infusion. In the team, the nurse evaluates and implements the intravenous therapy and selects the adequate materials for the patient and the treatment^5^. In the publications that we studied there was a greater likelihood for hemolysis with catheters at low gauges principally where there are high flows. This is due to the force of the blood with few lumes. Nevertheless, all of the researchers evaluated the infusion system as a whole being part of BIs with the catheter and not only the catheter in isolation^8^ ^,^ ^18^ ^,^ ^26^.

The technology is a part of everyday nursing. Progress in the use of BI in intravenous therapy has provided greater safety and efficiency in the process through the use of resources that facilitate and improve nursing in relation to: volume control, infusion time, memory of stunted infusions and the establishment of infusion pressure^4^.

The theme of safety for the patient is an important tool in the management of institutional processes and it has been receiving special attention in the world at large. In order to promote safety it is essential that all those involved understand clinical practices and techniques that are used to ensure low risks in transfusions. This being the case, an evaluation of the target public's characteristics is fundamental covering: age group, clinical state and blood component indicators. Other aspects include: variables related to devices and equipment, accuracy, influence of the hydrostatic pressure, solution type, the quality of the flux, system safety and evaluate costing issues. 

The following should be implemented and adhered to, in order to ensure transfusion safety: the development of protocols covering the parameters for uniformed evaluations and improvements in future equipment through better technology. For example, incentives should be given for the use of devices which are intelligent BIs that can alert the professional where there is the possibility of errors or when there are alterations in the safety standards. These devices are computerized and are thus connected to the patient's medical records when admitted to hospital. They can be adjusted to clinical needs and produce more efficiency in nursing. This is because notes can be directly placed on the electronic patient record as well as noting: the dose, time, type and volume of the medicine of infused solution^40^. This is technology that has been well developed and used in north American and European countries in spite of being expensive. Future studies covering cost and benefits for the implementation of intravenous therapy in relation to transfusion ought to focus on improving the process and work of nurses and the safety for patients at the edges of the hospital beds.

## Conclusion

The studies selected in the literature show that alterations in the integrity of the erythrocytes can occur when CH and ST are transfused by BI. Amongst the different types and BI mechanisms, the safest for transfusions are volumetric infusion pumps with the cassette mechanism. The linear peristaltic pumps are more likely to produce hemolytic effects. With reference to variable infusion speeds, based on the analyzed equipment, we found a divergence in the results.

We noted that the storage time for the blood components to be transfused can influence the increase in cellular fragility. In other words, the nearer the expiration deadline for the blood product was, the greater the chances of there being hemolysis. Red blood cell damage was mentioned in the publications when biomarkers were released with the most common being free hemoglobin due to potassium. The level of hemolysis was a determinant in some publications, which opted to designate the hemolysis from the reference value of 0.8%. None of the studies focused on the clinical consequences of extravascular hemolysis for the patient.

There was no conclusive evidence on the influence of the needle gauge with the catheter connected in the final infusion line, on the integrity of the erythrocytes for ST and CH. We opted to evaluate the whole infusion system for some of the publications. 

Investment in research and technology in relation to transfusion safety for the erythrocytes when using automated erythrocytes is extremely relevant. We need new research showing experiments that analyze the multi factors involved in hemolysis, which will in turn aid future studies that aim to promote safe practices for the protection of patients. 

### Limitations of the Study

We opted for not limiting the scope of the data and our searches, but some of the data was far from being relevant to the study. Although the theme was related to technology and innovation in transfusion therapy, there was a scarcity in publications on this topic covering the use of BIs in blood transfusions. 

Additional we could not evaluate the quality of the methods used in the in vitro experimental studies as they did not fit into the system for classifying data from epidemiological studies. They were considered to be pre-clinical. We therefore opted to describe the rigor in the methods used.
